# The Brain–Skin Axis in Psoriasis—Psychological, Psychiatric, Hormonal, and Dermatological Aspects

**DOI:** 10.3390/ijms23020669

**Published:** 2022-01-08

**Authors:** Luiza Marek-Jozefowicz, Rafał Czajkowski, Alina Borkowska, Bogusław Nedoszytko, Michał A. Żmijewski, Wiesław J. Cubała, Andrzej T. Slominski

**Affiliations:** 1 Department of Dermatology and Venerology, Faculty of Medicine, Ludwik Rydygier Collegium Medicum in Bydgoszcz, Nicolaus Copernicus University in Torun, 85-094 Bydgoszcz, Poland; r.czajkowski@cm.umk.pl; 2 Department of Clinical Neuropsychology, Collegium Medicum in Bydgoszcz, Nicolaus Copernicus University in Torun, 85-094 Bydgoszcz, Poland; alab@cm.umk.pl; 3 Department of Dermatology, Venereology and Allergology, Faculty of Medicine, Medical University of Gdansk, 80-210 Gdansk, Poland; boguslaw.nedoszytko@gumed.edu.pl; 4 Molecular Laboratory, Invicta Fertility and Reproductive Centre, 81-740 Sopot, Poland; 5 Department of Histology, Faculty of Medicine, Medical University of Gdansk, 80-210 Gdansk, Poland; mzmijewski@gumed.edu.pl; 6 Department of Psychiatry, Faculty of Medicine, Medical University of Gdansk, Debinki St. 7 build. 25, 80-952 Gdansk, Poland; cubala@gumed.edu.pl; 7 Department of Dermatology, University of Alabama at Birmingham, 500 22nd Street South, Birmingham, AL 35294, USA; aslomins@uthsc.edu; 8 Comprehensive Cancer Center, University of Alabama at Birmingham, 1824 6th Avenue, Birmingham, AL 35294, USA

**Keywords:** psoriasis, psychiatric disorders, psychological stress, psychoneuroimmunology, hormones

## Abstract

Psoriasis is a chronic inflammatory skin disease with systemic manifestation, in which psychological factors play an important role. The etiology of psoriasis is complex and multifactorial, including genetic background and environmental factors such as emotional or physical stress. Psychological stress may also play a role in exacerbation of psoriasis, by dysregulation of the hypothalamic–pituitary–adrenal (HPA) axis, sympathetic–adrenal–medullary axis, peripheral nervous system, and immune system. Skin cells also express various neuropeptides and hormones in response to stress, including the fully functional analog of the HPA axis. The deterioration of psoriatic lesions is accompanied by increased production of inflammatory mediators, which could contribute to the imbalance of neurotransmitters and the development of symptoms of depression and anxiety. Therefore, deregulation of the crosstalk between endocrine, paracrine, and autocrine stress signaling pathways contributes to clinical manifestations of psoriasis, which requires multidisciplinary approaches.

## 1. Introduction

Psoriasis is a chronic disease that significantly impairs the psychosocial functioning of patients, and it is recognized as a serious psychosomatic disease [1]. Distress related to the disease may lead to a significant decrease in quality of life [2], and in extreme cases may be a cause of depression or even suicide [3]. Patients with psoriasis are also at increased risk of other comorbidities including depression, anxiety, and suicidal ideation [4,5]. According to a cross-sectional multicenter study, suicidal ideation is increased in patients with psoriasis compared with the general population, and diagnosis of depression in patients with psoriasis remains inadequate [6]. Exacerbations of psoriasis episodes are often proceeded by stressful life episodes [7,8]. Psoriatic lesions are difficult to treat, especially when they occur on the hands or feet [9]. In patients with psoriasis, the occurrence of pruritus may be a biomarker associated with lack of response to therapy [10]. Systemic inflammation in psoriasis and deregulation of the circadian rhythm contribute to the failure of the peripheral nerve system [11]. Depression, a highly prevalent disease characterized by affective and cognitive disturbances [12] and even suicide ideation and attempts, is a significant factor in impairing the quality of life of patients with chronic diseases [13]. Increasing evidence has shown that psoriasis could lead to depression, and depression, in turn, exacerbates psoriasis, which may result in a vicious cycle of psoriasis and depression [14]. A study investigating 2391 psoriasis patients indicated that 62% of them had depressive symptoms [15]. Other studies have shown that depression generally predates psoriasis onset, and patients with moderate to severe depression have a significantly increased risk of psoriasis [16,17]. Therefore, there is a potential association between psoriasis and depression.

The skin actively responds to psychological stress, with involvement of skin immune cells, hormones and neurotransmitters [18,19]. Skin immune cells actively regulate tissue inflammation with their proinflammatory and anti-inflammatory effects. Stress-induced skin reactions primarily include cytokine secretion (e.g., interleukin-6 (IL-6), interleukin-1 (IL-1), interferon-γ (IL-γ)) and activation of skin peripheral corticotropin-releasing hormone (CRH) [18], proopiomelanocortin (POMC)-derived adrenocorticotropic hormone (ACTH), melanocyte-stimulating hormones (MSH) [20], and corticosteroid [21,22] production and activities, which counteract proinflammatory activities in a regulated fashion [23,24,25].

Interestingly, common skin stressors such as ultraviolet radiation also stimulate expression of the elements of the skin analog of the hypothalamic–pituitary–adrenal (HPA) axis, as well as other active neuropeptides including enkephalins and endorphins [19,24]. Furthermore, skin-produced neuropeptides may affect the part of the brain faction that forms the skin–brain axis [26]. It is necessary to note that ultraviolet radiation, including UVA in combination with psoralens (PUVA) or narrow band UVB (NB-UVB), is widely used in the treatment of psoriasis, and psoriasis can be characterized by seasonal changes in the severity of its symptoms, with the most severe symptoms presenting at the time of year with the lowest sunlight exposure [27]. It has also been established that ultraviolet light downregulates expression of proinflammatory cytokines, including IL-6, through activation of the skin analog of the HPA axis [23].

A meta-analysis showed that elevated levels of proinflammatory cytokines, as detected in patients with psoriasis, were noted in individuals with depression without comorbid systemic inflammatory diseases [28]. In similar studies, patients with depression were found to have elevated levels of proinflammatory cytokines, including tumor necrosis factor-α (TNF-α), interleukin 1β (IL-1β), IL-6, and C-reactive protein (CRP) [29]. These findings suggested that depression and psoriasis share a common diathesis rather than merely a psychosocial connection [17].

The nervous system, through the secretion of several inflammatory mediators, plays a key role in the pathogenesis of psoriasis. The cells of the immune system (lymphocyte, macrophage, mast cells) express a significant number of receptors for neurotransmitters and hormones, while the immune response can be modulated neurochemically [30,31]. Acute and chronic stress, anxiety, and depression affect innate and acquired immune responses, including an increase in the level of circulating proinflammatory cytokines, in particular interleukin IL-6 [32]. Serotonin, a neurotransmitter of the central and peripheral nervous system, being produced in the skin, represents a link between the nervous and immune systems and the skin [33,34,35]. Table 1 shows consequences brain–skin axis in patients with psoriasis.

The neuroendocrine system and the immune system also share many common mediators (e.g., neurotransmitters, neuropeptides, hormones, cytokines) and are interconnected by autonomic nerves and blood circulation [30,31,36,37]. The mononuclear cells (immunocytes) express receptors for neurotransmitters (acetylcholine, adrenaline, noradrenaline, and serotonin), neuropeptides (vasoactive intestinal peptide, substance P, and endorphin), and hormones (corticosteroids, prolactin, growth hormone, insulin, and sex hormones) [19,22,30,31,36,37,38]. During stress, changes in the levels of neuroendocrine mediators can modulate the activity of the immune system [18,22,30,31,36,39].

Conversely, cytokines secreted on the periphery in acute stress (from activated immunocytes) stimulate the adjacent afferent nerves or enter the CNS via blood circulation through the disrupted blood–brain barrier (stress-induced complex of reaction in the skin) [30,37,40]. Psoriasis is closely related to stress factors and emotional disorders, in which stress-dependent hormones increase serotonin synthesis, and therefore, the cutaneous serotonergic system and serotonin metabolism [33,35,41] may play a role in the pathogenesis of psoriasis [42].

**Table 1:** Brain–skin axis in patients with psoriasis.

Aspects	Consequences	Study
psychological	decrease in the quality of life	Randa et al. [2]
	stressful life episodes	Rousset et al. [8]
	cognitive impairment	Innamorati et al. [12]
psychiatric	anxiety, depression	Singh et al. [4], González-Parra et al. [14]
	suicidal ideation	Dalgard et al. [6]
hormonal, immunological	tissue inflammation, mood disorders	Slominski et al. [43], Kim et al. [44], González-Parra et al. [14], Ayasse et al. [45]
dermatological	exacerbates psoriasis	Kamiya et al. [46], Michalek et al. [7]

## 2. Psychological Stress, Inflammation of Psoriasis

Mental stress is a feeling of strain and pressure caused by internal perceptions that lead to anxiety or other negative emotions. Mental stress is commonly regarded as a well-established trigger of psoriasis, and many patients with psoriasis and physicians believe that mental stress exacerbates psoriasis [46].

Physiological responses to stress include increased sympathetic activity, over-activation of the HPA axis, and release of proinflammatory cytokines, which can, in turn, perpetuate and aggravate psoriasis, as the disease is associated with increased activity of several proinflammatory cytokines [47,48,49].

Inflammation may be a key factor in the explanatory model, since alterations of inflammatory modulators, such as the HPA axis and the sympathetic nervous system, are involved [50,51,52]. The HPA axis’ ability to regulate skin responses to stress and local immune activity has been described, and involvement of its malfunctioning in psoriasis has been suggested [25,43,53]. Psoriasis is an inflammatory disease mediated by T lymphocytes, with Th1 and Th17 profiles, and dendritic cells, which are activated and increased in the skin lesions. These cells (T lymphocytes, dendritic cells) seem to migrate to the skin and release inflammatory cytokines including interleukins 1 and 6 (IL-1, IL-6) and tumor necrosis factor alpha (TNF-α), which promote inflammation and proliferation of keratinocytes [54]. High levels of proinflammatory cytokines have been reported in major depression disorder and have shown an association with the severity of the illness. Psychological stress causes an increase in inflammatory markers, in particular C-reactive protein, TNF-α, IL-1β, and IL-6. In turn, exacerbation of psoriasis can increase comorbid depression and anxiety [49]. Acute stress has proinflammatory effects on the CNS in the way that it increases the permeability of the blood–brain barrier, which seems to be mediated through activation of CNS mast cells [55,56,57]. Therefore, in acute stress, IL-6 may cross the disrupted blood–brain barrier and activate the HPA axis [15,38,49]. This also concerns other mediators produced in the skin and activated by ultraviolet B (UVB) [26,58,59], a common skin stressor. IL-6 may induce lymphocyte activation, increase antibody production via CD4 T helper lymphocytes, and induce fever and acute-phase protein production. Keratinocytes also express the receptor for IL-10. Keratinocytes are thus both the effector and target cells for IL-10. IL-12 is secreted from human keratinocytes and has a systemic effect, inducing the Th1 immune response. The effects of IL-12 and IL-10 on keratinocytes are antagonistic, and their secretion depends on duration of psychological stress. Secretion of IL-12 is enhanced in short-term stress exposure, whereas secretion of IL-10 is enhanced in chronic stress [60].

A physiological mechanism that might be involved in this relationship is the activation of the local HPA axis [18,43]. It has been reported that CRH stimulates the synthesis of IL-6 and IL-11 in the skin and amplifies the expression of cell adhesion molecules in keratinocytes: HCAM, ICAM-1, major histocompatibility complex II, and HLA-DR, among others [18,44,61,62,63]. CRH activates the proinflammatory complex protein of the nuclear factor, NF-κB, which modulates DNA expression and immune responses in relation to stimuli including stress, ultraviolet light, free radicals, and infections [18,26,64]. These mechanisms activate the keratinocyte to a proinflammatory state, which can contribute to the development of psoriasis [53]. Biopsies from patients with psoriasis show a significant increase in CRH expression compared to healthy skin. This hormone is synthesized locally in the skin and hair follicles, or distributed through peripheral nerves [20,65], and would be a primary component of the brain–skin axis [18]. In those patients with stress-responsive psoriasis, acute anxiety may cause the production of inflammatory cytokines, without the proper release of anti-inflammatory cortisol to mitigate the skin’s response. Physiological levels of glucocorticoids (GCs) enhance the immune response by increasing the response of T lymphocytes to IL-2, promote the synthesis of cytokines including IL-1 and IL-6, and increase biological sensitivity to other cytokines, such as granulocytic colony stimulating factor, granulocytic and macrophage colony-stimulating factor, and interferon γ (IFN-γ) [46]. Site-specific immunological memory response in psoriasis has been linked to both CD8+CD103+ tissue resident memory T cells (Trm) and dendritic cells in the epidermis [66,67]. Trm cells may rapidly induce an inflammation, triggering the recurrence of the disease [67,68]. In psoriasis, they are represented by two main types: TRM CD8+CD69+CD103+ abundant in the lesional epidermis, and TRM CD4+CD69+CD103+ located in the dermis. CD103+ TRM cells produce IFN-γ, IL-17A, and IL-22. As regards CD8+ T lymphocytes, IL-17A is more commonly produced by TRM CD103+ cells than by CD103- T lymphocytes. Therefore, CD8+CD103+ TRM cells are effective in producing IL-17A [67,68,69]. Future research, aimed at better understanding epidermal Trm cell cytotoxic activity, will lead to the elucidation of their effects on the brain–skin axis and the development of advanced treatment strategies for psoriasis.

## 3. The Association between Psoriasis and Depression

Many inflammatory cytokines released in psoriasis are also released in depression, which suggests that there is a possible association between these diseases. The leading theory regarding pathogenesis of depression involves the dysfunction of several neurotransmitters, including monoamines (serotonin, norepinephrine, and dopamine), gamma-aminobutyric acid (GABA), and glutamate [70].

The content of serotonin, a key neurotransmitter that plays a role in the occurrence of psychoemotional disorders, was significantly reduced in patients with depression and anxiety–depressive states above the acute phase of inflammation factor. Increased serotonin levels, which also increase the risk of several anxiety disorders (for instance, social phobia), were observed only in psoriasis patients with associated anxiety disorders and also differed significantly from the control group. As for GABA, which is an inhibitory neurotransmitter, its decrease was noted in the majority of psoriasis patients with psychoemotional disorders. The obtained data indicate the need for a differential approach to the treatment of comorbid psychoemotional disorders in patients with psoriasis [71].

Studies have found increased expression of sIL6R in both depression and inflammation [72]. Furthermore, circulating sIL6R may cross the brain–blood barrier (BBB) and cause IL-6 trans-signaling in the CNS. IL-6, in its trans-signaling mode, increases the expression of indoleamine dioxygenase (IDO) in the CNS, resulting in a decrease in tryptophan levels and the production of tryptophan catabolites including kynurenine and quinolinic acid, which is related to depressive symptoms [73,74]. Harden et al. explored the expression of the tryptophan metabolism enzyme L-kynureninase (KYNU) in psoriatic human skin, normal human skin, blood cells, and primary cells and found KYNU+cells in psoriatic lesional cells, their expression being positively correlated with disease activity [75]. Growing studies have shown that increased levels of kynurenine, quinolinic acid, and IL-6 have all been found in patients with depression [76]. Th17 cells participate in depression, which is activated by several cytokines, such as IL-1β, TNF-α, and IL-6. In contrast, depression can increase the level of proinflammatory cytokines, which can result in or exacerbate psoriasis [77]. Cytokine stimulation of the HPA axis may also be a factor in depression pathophysiology, as hypersecretion of CRH is associated with depression [78]. Interleukin-1, IL-6, TNF-α, and IFN-α increase levels of CRH, adrenocorticotropic hormone (ACTH), and cortisol, which are all activators of the generalized stress response [45,79]. These cytokines also decrease the expression and activity of glucocorticoid receptors, which downregulates the negative feedback loop of the HPA system, further increasing cortisol release [80,81,82,83,84]. Overactivation of this system promotes negative mood symptoms. Emerging research suggests that there may be a physiological link between psoriasis and depression. Proinflammatory cytokines including TNF-α IL-12, IL-17, IL-23, and IFN-γ are elevated in psoriasis. Patients with depression often have elevated TNF-α, IL-1, IL-1β, IL-2, IL-6, IL-8, IL-17, IL-21, IL-23, C-reactive protein, and TGF-β [70,85,86].

In addition, anxiety is associated with activation of the sympathetic nervous system, which may have a role in the autonomic modulation and in the inhibition of the parasympathetic system. Patients suffering from depression report an increased sympathetic tone, at rest and during a stressful task, which is evidenced by the increase in serum markers, especially plasma norepinephrine. The sympathetic system also innervates primary and secondary lymphoid organs and promotes inflammation, with an increase in IL-6 and IL-1β [49,87]. The alpha-adrenergic receptors of the sympathetic system seem to be involved in the increase in cytokines and proinflammatory changes, which would lead to triggering or maintaining an outbreak of psoriasis. Norepinephrine released during stress activates these receptors in macrophages and dendritic cells, leading to an increase in TNF-α and suppression of anti-inflammatory IL-10 [88].

## 4. The HPA Axis Links Psoriasis and Depression

The HPA axis is one of the main components of the neuroendocrine system which, by secretion of a series of hormones and complex feedback mechanisms, coordinates interaction between major endocrine organs: the hypothalamus, pituitary gland, and adrenal glands [89,90].

**Figure 1:**
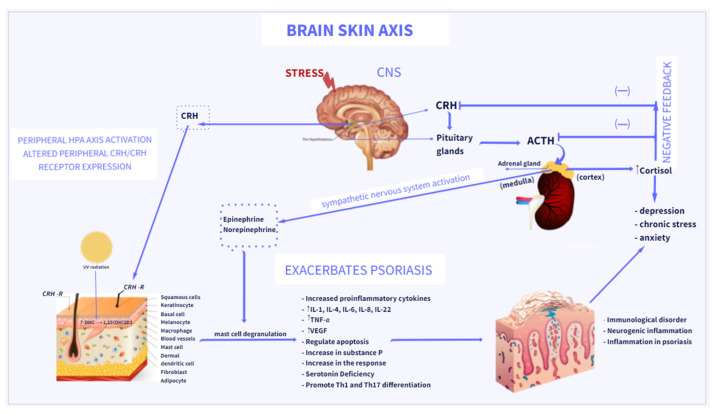
Brain–skin axis: association between psoriasis and depression. Stress acts via several pathways to exacerbate psoriasis, via the central and peripheral HPA axes, regulation of cytokine production, and activation of the sympathetic nervous system. The final messengers of the sympathetic nervous system and HPA, norepinephrine and cortisol, can directly engage in regulation of various immune cells to modulate immune responses. Direct action of CRH induces inflammatory responses in psoriasis. CRH and CRH-related peptides can be produced by several cells in the skin and stimulate the local production of cytokines in the skin. By binding to CRH-R on mast cells, CRH induces mast cell degranulation and releases proinflammatory factors, which induces further inflammation in psoriasis. The increase in cortisol levels causes the exacerbation of psoriasis and the activation of Th-17 cells, which leads to an increase in the levels of pro-inflammatory cytokines IL-17, TNF-α contributing to the development and intensification of depressive disorders. Vitamin D_3_ (Vit D_3_) is synthesized in the skin from its precursor 7-DHC under the influence of UVB and metabolized to its active form, 1,25(OH)_2_D_3_.

An activation of the HPA axis is a major mechanism of the body’s response to stress [18,89,90]. Acute or chronic stress activates the HPA axis, resulting in the production of CRH by the paraventricular hypothalamic nucleus; ACTH by the anterior pituitary gland; and ultimately glucocorticoids (cortisol or corticosterone), by the adrenal cortex [43,89,90] (Figure 1). An increasing number of studies has shown that the HPA axis could participate in the development of psoriasis and depression. CRH plays a central role in psoriasis [18,53,91]. Skin has a fully functional peripheral HPA system that contributes to the interaction between skin and brain by releasing hormones including CRH, ACTH, and GCs [43,92]. The crosstalk between the brain and the skin is called the brain–skin axis [18,19], and provides a link between psoriasis and depression. An induction of skin inflammation results in activation of a skin equivalent of the HPA axis by various proinflammatory cytokines produced locally. Furthermore, locally expressed stimulatory signals may induce the central HPA axis [23,26,93]. CRH is mainly released in the CNS, which plays a key role in orchestrating the HPA axis and affects the cutaneous immune system. In the skin, CRH can be produced by several peripheral cells, including sebocytes, fibroblasts, melanocytes, keratinocytes, and mast cells [65]. Moreover, CRH has been found to stimulate the secretion of proinflammatory cytokines, through the interaction with CRH-receptor type 1 (CRHR1) [18,19]. CRHR1 can also be activated by the CRH-related urocortins that are expressed in the skin [94,95]. By stimulating CRHR1, CRH activates diverse signaling pathways that regulate apoptosis, proliferation, differentiation, and anti- or proinflammatory activities of skin cells [61,96,97,98]. Furthermore, evidence has shown that psoriatic skin lesions have a higher expression of CRHR1 than healthy skin [99]. Skin mast cells are regarded as the “central switchboards” of neuroinflammation and play a key role in skin stress responses [56,57,100]. Studies have shown that mast cells can express CRHR1 in close proximity to psoriatic plaques, which contribute to the degranulation of mast cells induced by CRH, increasing vascular permeability and exhibiting proinflammatory functions. The expression of CRHR1 and CRH induced mast cell secretion of IL-6, IL-8, IL-22, and vascular endothelial growth factor (VEGF), which participate in the pathogenesis of psoriatic lesions. In turn, IL-6 secreted by mast cells can induce CRH secretion by activating the HPA axis [45]. Interestingly, a recent study has shown deregulation of the skin equivalent of the HPA axis in cutaneous mastocytosis (neoplastic proliferation of mast cells in the skin) [101], which highlights the underlying importance of mast cells in dermal homeostasis.

Two major types of receptors can help the HPA axis exert its feedback function: the glucocorticoid (GR) and mineralocorticoid receptor (MR) [102]. MR has a higher affinity for cortisol than other ligands. However, it has low specificity and binds both aldosterone and cortisol. GR binding is highly specific for cortisol but responds to higher concentrations than MR, and MR is more likely to take effect during acute or normal stress. However, GR is more easily activated in severe or prolonged stress. Moreover, increased sensitivity of the adrenal glands to ACTH has also been observed in patients with depression, which will contribute to the release of glucocorticoids. Glucocorticoids have proinflammatory and anti-inflammatory effects [103]. Accumulating evidence indicates that glucocorticoids can suppress their anti-inflammatory effects and exhibit proinflammatory properties under acute or chronic stress. IL-6 works with IL-1β to induce a systemic immune response, promoting psycho-neuro-immunological changes in depressed patients [16,77].

The local skin axis of CRH–POMC–ACTH corticosteroids is important for the skin’s response to stress. Keratinocytes and dermal fibroblasts secrete CRH, which binds the appropriate receptors (CRH-R1), thus stimulating them to produce POMC, whose degradable peptide hormones (ACTH, MSH, and β-endorphin) also bind their corresponding receptors [18]. Under the influence of the locally increased secretion of ACTH, dermal fibroblasts secrete corticosterone; thus, the skin has a functional connection with the CRH–POMC–ACTH–corticosterone axis [40,43,104,105].

## 5. Neuropeptides in Psoriasis and Depression

Cutaneous denervation leads to significant improvement and remission of psoriasis, indicating the significant pathogenic roles of neuropeptides in psoriasis [106]. If psychosocial stress exacerbates psoriasis, then mast cells are likely to be of pivotal importance. In healthy skin, they play a central role in the cutaneous response to stress and can be considered as “central switchboards” of neurogenic inflammation. Several stress-related neuropeptides and neurotrophins are closely involved in this response and act as mast-cell secretagogues, including CRH, substance P, CGRP, and nerve growth factor [55,107]. In psoriasis, mast cells located in the upper dermis serve pleiotropic functions in psoriatic plaque evolution [44]. Early lesions show multiple degranulated mast cells, whereas chronic lesions demonstrate multiple activated mast cells producing inflammatory mediators [108]. Despite recent advances [109], the precise role of mast cells in the psychosocial stress–psoriasis relationship is still unclear and merits further research.

Neuropeptides regulate various physiological functions including learning, memory, sleep disorders, and pain [110,111]. Neuropeptides can be excitatory or inhibitory, and they affect the amount and type of neurotransmitter release in stimulus responses (acting as neuromodulators). Neuropeptides can also act as neurotransmitters, as hormones or endogenous opioids that suppress the sensation of pain and arouse the sense of comfort, as well as immunomodulators. Not only do nerve cells secrete numerous neuropeptides (called neurokinins) and neurotransmitters, but keratinocytes, in response to various stimuli, are also able to produce them (e.g., adrenaline, noradrenaline, dopamine, histamine, acetylcholine, nerve growth factor, and substance P) [112,113]. Keratinocytes can also express receptors for the aforementioned neurotransmitters, neurotrophins, and neuropeptides, which are important in linking psychoneuroimmunological mechanisms [19,91]. Along with keratinocytes, fibroblast functions are also affected by the nervous system, for example, the impact of epinephrine on migration and collagen production (important steps in wound healing) [114].

As the skin is highly innervated, peripheral nerves can impact skin homeostasis by releasing neuropeptides including substance P (SP), brain-derived neurotrophic factor (BDNF), and nerve growth factor (NGF), which serve as local stress responders that mediate neurogenic inflammation. SP is a stress-related proinflammatory neuropeptide. The biological effect of SP is mainly mediated by neurokinin (NK)-1 receptors, since SP is the natural ligand with the highest affinity for NK-1 receptors. The SP-NK-1 receptor pathway can be activated in response to stressful stimulation in both the peripheral nervous system (PNS) and the CNS. SP promotes the proliferation of T lymphocytes, and most immune cells that produce SP can also express NK-1 receptors. Double staining showed that NK-1 receptor-positive cells were predominantly mast cells, suggesting an important role of NK-1 receptor activation in mast cell degranulation induced by stress. Other studies indicated that SP could participate in the effect of CRH on the degranulation of mast cells during stress [115]. In addition, SP causes the infiltration of inflammatory cells, including macrophages and neutrophils, and leads to monocytes and T cells releasing various cytokines, especially IL-12, IL-1, and IL-6 [116]. SP also enhances the survival of dendritic cells by inhibiting the apoptosis of bone-marrow-derived dendritic cells. In summary, the proinflammatory mechanism of the SP-NK-1 receptor pathway plays a key role in the progression of psoriasis. In depression, high levels of SP have also been observed, suggesting that the pathological mechanism of depression is associated with malfunction of SP/NK-1-mediated responses [117].

BDNF plays a crucial role in central nervous system (CNS) development and is involved in neuroprotective and neurodegenerative processes. BDNF is a neurotrophin that is involved in brain functions including learning and memory. The specific receptor of BDNF is tyrosine kinase receptor B (TrkB) [118]. It has been found that a decrease in BDNF levels is common in psoriasis and depression, which is a possible factor linking psoriasis with depression [119]. In addition, an increasing number of studies have reported that there is an association between BDNF/TrkB signaling and the 5-HT system, and this interaction is possibly the mechanism by which BDNF influences depressive behaviors [120,121]. Given the central role played by BDNF/TrkB signaling in cell function, it is not surprising that changes in expression, traffic, and/or stability of this neurotrophin and its high-affinity receptor are common mechanisms for many human pathologies [122]. NGF is released at high concentrations during inflammation and mediates cutaneous reinnervation. Keratinocytes, fibroblasts, nerves, and adipocytes secrete NGF (nerve growth factor) [123]. By regulating neurogenic inflammation, NGF plays important roles in the development of psoriasis and is related to the intensity of pruritus. NGF binds with high affinity to TrkA, TrkB, and TrkC and induces neuroinflammation by promoting mast cell degranulation [124]. NGF can recruit and activate T lymphocytes to promote the inflammatory response in psoriasis. Other neuropeptides, including SP, which are probably implicated in psoriatic pathogenesis, are regulated by NGF [125]. However, other studies have shown that, compared to healthy individuals, low levels of NGF were found in the hippocampus in patients with depression, which is contradictory to the high concentration of NGF in psoriasis [126,127]. In future research, the extra role of NGF in the development of depression and psoriasis should be clarified.

## 6. Sunlight Deficiency Influences Psoriasis and Depression

As mentioned above, sunlight deficiency strongly affects the severity of skin manifestation of psoriasis [128]. Sun exposure, as well as PUVA or NB-UVB, is a widely accepted form of treatment of psoriasis. However, potentially important adverse effects of “light therapy”, including skin aging [129] and skin cancer [130], should be carefully considered. On the other hand, seasonal lack of sunlight observed at higher latitudes (e.g., Northern Europe) is believed to be the main factor in the development of depression [131]. The sun, or rather the lack of sun, could be a causative factor in the development of psoriasis as well as depression with several common manifestations. Interestingly, the UVB fraction of sunlight is also required for skin formation of vitamin D, and the skin cells also possess fully functional enzymatic machinery to convert vitamin D to its active metabolite–calcitriol [25,132,133,134]. The skin cells (keratinocytes) also express vitamin D receptor (VDR), and the skin is a very important target for vitamin D metabolite activity [135]. Alternative nuclear receptors for vitamin D metabolites have recently been reported to be expressed in the skin. Vitamin D not only modulates or suppresses inflammation in psoriasis, it also rectifies the abnormal epidermal function related to this condition [136,137,138]. It was demonstrated that deletion in late cornified envelope genes, LCE3B and LCE3C, located within PSORS4 is a genetic risk factor of psoriasis, suggesting disruption of the differentiation process in psoriasis [139]. Furthermore, calcitriol together with calcium regulates proliferation and differentiation of keratinocytes as well as activity of the cutaneous immune system [140,141]. Vitamin D deficiency is an important factor in the development and progression of psoriasis [142,143]. Consequently, vitamin D and its analogs are considered as important factors supporting eradication of psoriatic plaques [144,145]. Several studies have proved that vitamin D analogs could be successfully used as supplements to compensate for vitamin D deficiency [146,147]; they could also be used directly through topical application [148,149]. Recently, high doses of vitamin D were found to be efficient in eradication of psoriatic plaques [150]. In addition, the efficiency of co-treatment of psoriatic patients with vitamin D and glucocorticoids was recently supported by several clinical studies in combination with calcipotriol and betamethasone dipropionate [151,152]. There is ongoing debate about whether vitamin D levels also influence mood, behavior, learning, and other brain functions. Most importantly, active forms of vitamin D regulate the expression of neurotrophins, including neural growth factor (NGF) and neurotransmitters (acetylcholine, dopamine, and gamma-aminobutyric acid) [153]. Calcitriol was also found to stimulate the gene expression of tyrosine hydroxylase, which is considered to be a rate-limiting step in the synthesis of the catecholamines. These neurotransmitters (dopamine, noradrenaline, and adrenaline) are implicated in the pathophysiology of mood disorders. If there is a causal relationship whereby vitamin D insufficiency or deficiency provides a risk to later depression [154], shortening sun exposure is associated with an increased risk of depression [155].

In addition to neuropeptides, skin-derived vitamin D could form a direct link be-tween the skin and the brain, as its deficiency is common in both psoriasis and depression.

## 7. Conclusions

The relationship between mental stress and the clinical course of psoriasis is com-plex and still not fully understood. The pathophysiological mechanisms suggest a role for nerve-related factors, namely, their interaction with mast cells and the severity of neurogenic inflammation in this regard. Changes in the HPA axis and sympathetic–adrenal–spinal malfunction testify to the differences between patients with psoriasis and healthy subjects in response to stress. Stress redistribution with increased transport of leukocytes into the skin can exacerbate psoriasis. The severity of psoriatic lesions contributes to the self-isolation of the patient and the development of depressive disorders in some patients. Thanks to the use of nanotechnology in the treatment of psoriasis, it will be possible to increase the efficiency of topical drug delivery and limit the systemic use of immunosuppressive drugs [156,157,158], especially during pandemics.

## Data Availability

Not applicable.
